# Family medicine and general practitioner supervisor wellbeing: a literature review

**DOI:** 10.3399/BJGPO.2023.0230

**Published:** 2024-07-10

**Authors:** Shaun Prentice, Helen Mullner, Jill Benson, Margaret Kay

**Affiliations:** 1 School of Psychology, The University of Adelaide, Adelaide, South Australia; 2 General Practice Training Research Department, Royal Australian College of General Practitioners, Adelaide, South Australia; 3 General Practice Training, Royal Australian College of General Practitioners, Adelaide, South Australia; 4 School of Medicine, The University of Adelaide, Adelaide, Australia; 5 General Practice Clinical Unit, Faculty of Medicine, The University of Queensland, Brisbane, Queensland

**Keywords:** postgraduate education, education, burnout, psychological, doctors' health, systematic reviews, general practice

## Abstract

**Background:**

Research examining general practice supervisor wellbeing has often been conducted within the context of trainee wellbeing and educational outcomes.

**Aim:**

To consolidate the current literature regarding the wellbeing of GP supervisors through a ‘supervisor–wellbeing’ lens.

**Design & setting:**

Literature review of original research studies on Embase, Ovid MEDLINE, and Ovid PsycINFO from inception to December 2022.

**Method:**

The Embase, Ovid MEDLINE, and Ovid PsycINFO databases were systematically searched from inception to December 2022. Original research studies were eligible if they explored any aspect of wellbeing or burnout (that is, construct conceptualisations, risk and protective factors, implications, or interventions) among GPs involved in educating GP trainees. Reporting quality of included studies was assessed using the QualSyst tool. Results from included studies were narratively synthesised.

**Results:**

Data from 26 independent samples were reviewed. Burnout was generally conceptualised using the Leiter and Maslach model. Wellbeing was poorly defined in the literature, largely being conceptualised in personal psychological terms and, to a lesser extent, professional satisfaction. Risk and protective factors were identified and grouped as individual (for example, satisfaction with capacity to teach) and external (for example, autonomy, collegial relationships, resource availability) factors. GP supervisors’ wellbeing appeared to affect their job performance and retention. This review identified only two studies evaluating interventions to support GP supervisors’ wellbeing.

**Conclusion:**

The present review highlights a lack of conceptual clarity and research examining interventions for GP supervisor wellbeing. It provides guidance for future research designed to maximise the wellbeing of GP supervisors and support the wellbeing of trainees.

## How this fits in

Little research has considered the wellbeing of GP supervisors. Using a ‘GP supervisor lens’, this literature review identified research gaps in construct conceptualisations, the consequences of supervisor wellbeing or burnout, and interventions for this group. Research exploring risk and protective factors has focused on individual factors rather than organisational and systemic contributors. This review highlights suggestions for future research directions to support GP supervisors’ wellbeing.

## Introduction

The health and wellbeing of the clinicians supervising postgraduate medical trainees is rarely discussed. Yet this matter is fundamentally important. Beyond the clinical and financial implications of poor wellbeing for this group,^1,2^ their educational role means supervisors’ wellbeing can affect trainees’ wellbeing^3–7^ and professional identity formation,^4,5,8^ and educational quality.^9–11^ Research has highlighted how the wellbeing of GP supervisors (sometimes referred to as 'GP faculty') can impact trainees (especially concerning teaching quality)^9–12^ but less consideration has been given to supervisor wellbeing in its own right. Given increased demands on supervisors to support trainee wellbeing,^13^ the medical education community has a duty to support its supervisors.

This systematic literature review used a supervisor–wellbeing lens to capture the breadth of research in this field. It focused on understanding how GP supervisors conceptualised wellbeing and burnout, risk and protective factors, and the consequences of both poor and excellent health. Interventions designed to address GP supervisor health were also sought.

## Method

Research questions are presented in Box 1. The Preferred Reporting Items for Systematic reviews and Meta-Analyses (PRISMA) 2020 statement was adhered to.^14^ For this study, the authors viewed burnout and wellbeing as discrete states that lay on the same continuum,^15^ but did not explicitly define them given their interest in understanding how they were conceptualised in the literature. However, the authors acknowledged the absence of burnout as a necessary, but insufficient, condition for wellbeing.^16^

Box 1 **Research questions for the systematic review**

**Table:** 

How are wellbeing and burnout conceptualised in the GP supervisor wellbeing literature? What are the risk factors and protective factors for GP supervisor wellbeing and burnout? What are the consequences of poor and excellent GP supervisor wellbeing? What interventions have been proposed or implemented to address GP supervisor wellbeing?

### Search strategy and study eligibility

SP, in consultation with JB, generated a comprehensive search strategy by combining terms relating to wellbeing (for example, wellbeing, burnout, stress, resilience), family medicine and general practice, and medical supervisors (including educators and faculty). Logic grids were prepared for Embase, Ovid MEDLINE, and Ovid PsycINFO (see Supplementary Information S1).

For inclusion, studies needed to meet all four eligibility criteria. First, studies needed to explore any aspect of wellbeing relating to any research question (that is, conceptualisation, risk or protective factors, consequences, or interventions). Wellbeing was broadly construed to include physical, psychological, and social wellbeing, plus professional attributes (for example, job satisfaction). Second, participants needed to be GP supervisors, defined as primary care generalist medical practitioners responsible for supervising and/or delivering education to GP trainees. Internationally, the duties of GP supervisors varies. For instance, in vocational models (for example, Australia), GP supervisors balance clinical responsibilities with teaching, whereas in the USA, family medicine faculty they also tend to conduct research.^17,18^ GP supervisors responsible for managing training programmes (that is, programme directors) were included. Third, studies needed to report on primary or secondary data (that is, not opinion or commentary pieces). Finally, articles needed to be published in a peer-reviewed journal with an English version available.^19,20^ Conference abstracts were excluded to maximise the quality of included data, and grey literature was excluded.

Database results were imported into EndNote (version 20). Duplicate records were removed and remaining results screened.^21,22^ SP subsequently conducted citation 'pearl-growing' (via Scopus) and citation searching of all included articles. Database alerts were established and monitored until December 2022. To establish inter-rater reliability of the screening process, HM independently screened a random sample of titles and abstracts (10%, N^studies^ = 58) and citations shortlisted for full-text screening (33%, N^studies^ = 20). Inter-rater reliability for both phases of screening was excellent at 82.8% and 90%, respectively.

### Data extraction

Full texts of included articles were imported into NVivo (version 12; see Supplementary Information S2 for attributes extracted). Studies’ samples were evaluated for overlap and collated into one NVivo case where this occurred. SP reviewed each article line-by-line, storing information within codes corresponding to the research questions. Only content within the results section was coded. However, content pertaining to conceptualisations was also drawn from articles’ introduction and method sections.

### Assessment of article reporting quality

To inform the weighting of studies’ findings, SP and HM independently assessed studies’ reporting quality using the QualSyst tool.^23^ Final inter-rater reliability and agreement were 74.29% and 100%, respectively.

### Data synthesis and analysis

Given the breadth of the research questions, SP narratively synthesised findings by reviewing the content within each node for similar ideas (for example, all content regarding psychological traits was grouped). SP then collated these groupings into overarching subject categories.

## Results

### Study screening and characteristics

**Figure 1.**
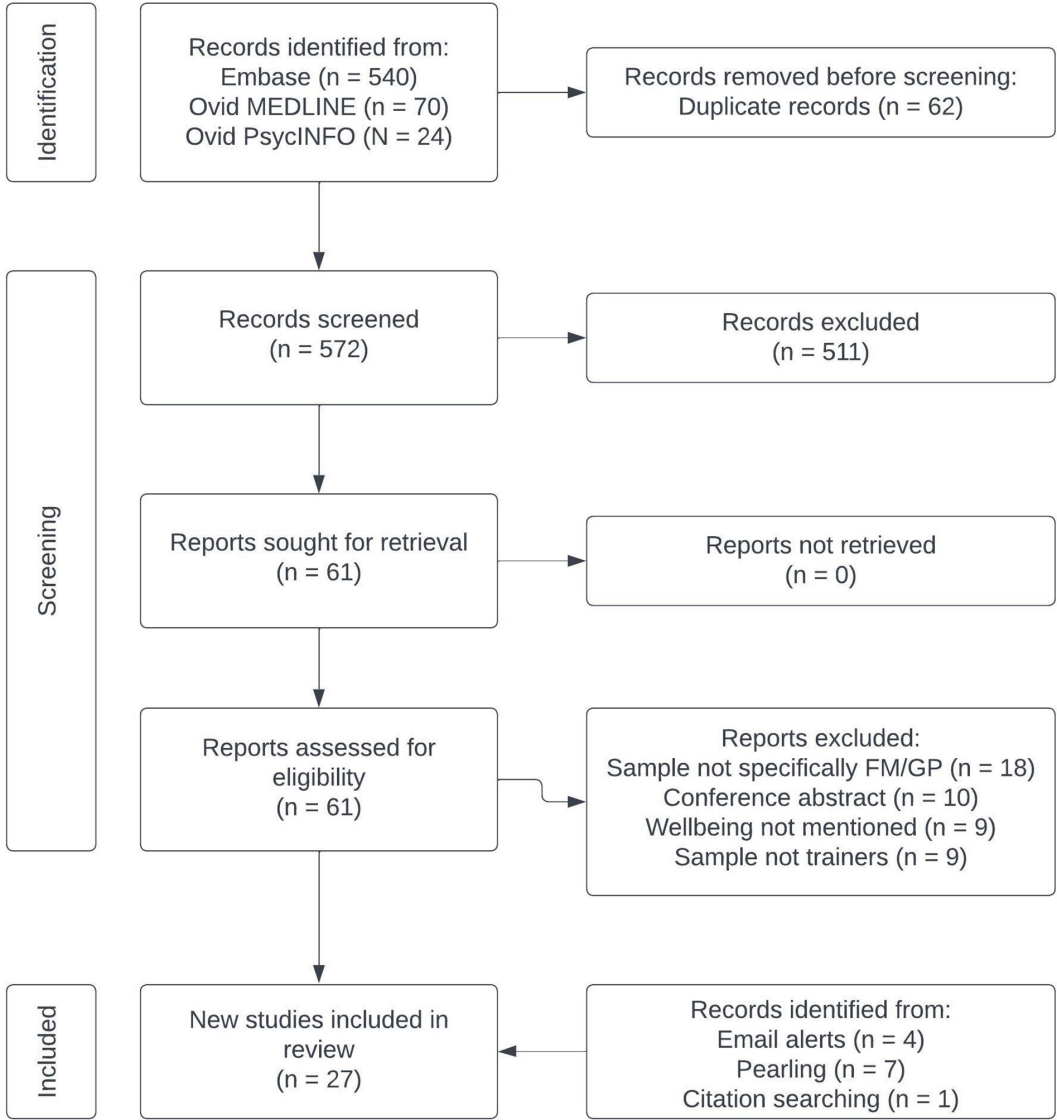
PRISMA diagram for selection of eligible studies.^14^ FM = family medicine

Figure 1 depicts the screening process. Supplementary Information S3 details records excluded at the full-text stage. Two studies used overlapping samples,^24,25^ so both studies were coded within one case (that is, treated as one sample). Ultimately, 26 unique samples were included (see Table 1).^17,24–49^

**Table 1.** Characteristics of 27 included studies examining wellbeing among family medicine and general practice supervisors

Citation		Study approach			Sample details			
	Publication year	Methodology	Method	Design	Data collection year	Sample country	Sample size	Response rate
Adil Al-Sulaiman and Abdul-Rahman Al-Bunaian^27^	2021	Quantitative	Survey	Cross-sectional	NR	Saudi Arabia	32	64%
Agana *et al* ^26^	2017	Qualitative	Focus Groups	Cross-sectional	NR	US	26	87%
Al-Saab *et al* ^28^	2022	Quantitative	Survey	Cross-sectional	2020	Saudi Arabia	80	93%
Awadallah *et al* ^29^	2021	Quantitative	Survey	Cross-sectional	2020	US	151	NR
Buck *et al* ^30^	2019	Quantitative	Survey	Cross-sectional	2017	US	116	NR
Chambers and Campbell^31^	1996	Quantitative	Survey	Cross-sectional	1994	England	77	NR
Coenen *et al* ^32^	2022	Multiple methods	Survey	Cross-sectional	2020	Belgium	311	26%
Cohen-Katz *et al* ^33^	2003	Qualitative	Focus groups, interviews, participant observation	Longitudinal	NR	US	NA	NA
Costa *et al* ^34^	2005	Quantitative	Survey	Cross-sectional	2002	US	1418	47%
Ferber *et al* ^24^ and Jacobs *et al* ^25^ ^a^	2022 (both)	Quantitative	Survey	Cross-sectional	2020	US	862	20%
Fernald *et al* ^35^	2021	Qualitative	Interviews	Cross-sectional	2020	US	25	NA
Garr^36^	1986	Quantitative	Survey	Cross-sectional	1985	US	695	71%
Kay and D'Amico^37^	1999	Quantitative	Survey	Cross-sectional	1997	US	383	59%
Ko, Guck^17^	2020	Quantitative	Survey	Cross-sectional	2017	US	103	53%
Krueger *et al* ^38^	2017	Quantitative	Survey	Cross-sectional	NR	Canada	687	67%
Levy *et al* ^39^	2018	Qualitative	Interviews	Cross-sectional	NR	Canada	13	NA
Locke *et al* ^40^	2020	Quantitative	Pre-post survey	Longitudinal	2017	US	52	96%
Longenecker *et al* ^41^	1997	Quantitative	Survey	Cross-sectional	1975	US	240	NR
Meurer *et al* ^42^	1998	Quantitative	Survey	Cross-sectional	1995	US	399	80%
Nutting *et al* ^43^	2021	Multiple methods	Pre-post survey	Longitudinal	NR	US	12	50%
Ofei-Dodoo *et al* ^44^	2018	Quantitative	Survey	Cross-sectional	2017	US	307	72%
Porter *et al* ^45^	2018	Quantitative	Survey	Cross-sectional	2016	US	245	54%
Probst *et al* ^46^	1998	Quantitative	Survey	Cross-sectional	1995	US	69	87%
Psenka *et al* ^47^	2021	Quantitative	Survey	Cross-sectional	2019	US	268	45%
Purdy *et al* ^48^	1987	Quantitative	Survey	Cross-sectional	1984	US	18	100%
Simpson *et al* ^49^	2001	Qualitative	Interviews	Cross-sectional	NR	US	24	NA

NA = not applicable. NR = not reported.

^a^Two studies used overlapping samples, so were coded within one case.

Among the 21 quantitative studies included, details regarding samples, analysis, and results were generally well-reported. However, the surveys were not always adequately described, particularly when using custom-designed questions. Only nine studies (47%) provided variance estimates for the main results. The two quantitative interventional studies gave limited consideration to the effects of confounders. The reporting quality for the seven studies with qualitative data was generally high. Nevertheless, only three studies clearly described the study’s context, while four did not discuss reflexivity. Supplementary Information S4 provides further details concerning reporting quality.

### How burnout and wellbeing are conceptualised

Burnout was overwhelmingly conceptualised using the Leiter and Maslach model of burnout, comprising emotional exhaustion (feeling emotionally drained), depersonalisation (becoming detached from one’s work and patients), and low personal accomplishment lacking a sense of achievement in one’s work).^17,24,29,30,38,40,43–45,47,48,50–54^ One study offered a novel definition of burnout,^48^ but this was not based on empirical data. Wellbeing was not explicitly defined, but facets were measured using several instruments (see Supplementary Information S5 for a list). These facets were organised using a model developed for GP trainees, which describes connected personal and professional domains.^55,56^ Within supervisors’ personal domain, researchers focused on psychological wellbeing, particularly resilience;^30,45^ stress;^27,29,38^ and mental health.^25,29,38^ Fewer studies considered physical (adequate sleep;^30^ and global self-reported health^27,29,38^) or social wellbeing (loneliness^47^). Supervisors’ professional wellbeing was predominantly operationalised through job satisfaction^26,27,37,38,41,42,44,46^ and meaningfulness.^25,35,42^ One study assessed supervisors’ professional relationships and commitment to their job.^25^ Personal–professional interactions and value fulfilment were noted in one study each.^26,30^

### Risk and protective factors for supervisors’ wellbeing

Most studies focused on risk and protective factors for GP supervisor wellbeing. These were coded as internal factors (that is, personal factors; skills and abilities; and healthcare role) and external factors (that is, learning and practice environment; organisation; the regulatory, business and payer environment; and sociocultural factors).^57^ Factors identified are listed in Table 2.

**Table 2.** Overview of risk and protective factors for GP supervisors’ wellbeing described in the literature organised using Brigham *et al*’s^57^ model

Domain	Category	Risk factors	Mixed evidence	Protective factors
Internal	Personal	- Emotional exhaustion.- Depersonalisation.- Stressful personal responsibilities.	- Sex.- Relationship status.- Age.- Minority group membership.- Psychological stress.	- Self-reported health status.- Engagement in positive health behaviours.- Psychological flexibility.- Resilience.- Satisfaction with personal responsibilities.
	Skills and abilities			- Ability to disengage from work.- Capacity to balance competing demands.
	Healthcare role		Duration in current position	- Clinical variety.- Satisfaction with frequency of on-call duties.- Patient interactions.- Teaching.- Sense of meaningfulness.- Duration of involvement in medical education.- Satisfaction with balance of duties.
External	Learning and practice environment			- Peer interaction.- Autonomy.- Satisfaction with resourcing.
	Organisation	High workload	Programme change	- Satisfaction with leadership.
	Regulatory, business and payer environment			- Acknowledgement.- Satisfaction with hospital credentialing policies.
	Sociocultural		COVID-19 pandemic	

### Internal factors

The focus of most studies was on personal factors, although the effect of these factors were mixed. Females generally displayed lower job satisfaction and higher depressive symptoms,^34,36,38^ but also lower emotional exhaustion and higher personal accomplishment levels.^30^ Other studies found no sex differences,^45,47^ with one study noting that professional experience and other sociodemographic factors diminished sex differences.^36^ Similar mixed findings were observed for other personal factors including relationship status, age, and minority group membership.^24,25,28,30,34,38,47^

Greater consistency emerged for health status and psychological traits. Self-reported health status and engagement in positive health behaviours positively correlated with job satisfaction and negatively correlated with burnout.^30,38^ Similarly, psychological flexibility (that is, being able to adapt one’s behaviours to align with valued goal pursuit)^58^ predicted higher personal accomplishment and resilience, while resilience buffered against burnout.^30^ Higher stress predicted higher depression levels and burnout, but also greater job satisfaction.^27,34,38^ Conversely, higher emotional exhaustion and depersonalisation predicted lower job satisfaction.^27,38^ Stressful personal responsibilities adversely affected supervisors’ ability to integrate their work and personal lives,^34,39,45,49^ whereas satisfaction in this domain was associated with greater resilience.^45^

Skills and abilities received limited attention, focusing on coping strategies. Ability to disengage from work in one’s personal domain protected against emotional exhaustion and depersonalisation, and positively correlated with resilience.^45^ Likewise, new GP supervisors reported that their ability to balance competing demands moderated how overwhelming they found the transition into their role.^39^

More studies focused on supervisors’ roles. Individuals’ clinical responsibilities were key, with supervisors identifying clinical variety as appealing.^26^ Similarly, satisfaction with frequency of being on-call for clinical duties strongly predicted overall job satisfaction (OR = 6.2).^37^ Patient interactions could be opportunities for growth and a source of vitality.^26,49^ Likewise, teaching was highly valued and seen as protective.^26,34,37,49^ Related to these factors was one’s sense of professional meaningfulness. GP supervisors who desired to teach and felt they were achieving their goals reported higher job satisfaction.^37,46^ Similarly, satisfaction with the balance between their time for patient care and other residency duties was protective.^17,37^ Although duration in one’s position inconsistently related to wellbeing,^28,35,44,45^ the longer one was involved in medical education, the greater one’s job satisfaction and professional accomplishment.^42^

### External factors

Most studies considering external factors focused on the learning and practice environment.^57^ Peer interaction was crucial for wellbeing, reducing the burden borne by any individual.^26,35,49^ Satisfaction with teamwork and collegial relationships protected against emotional exhaustion and depersonalisation, and promoted engagement with teaching.^25,47^ Although the effect of satisfaction with collegial interactions on job satisfaction was unclear,^27,37,38^ satisfaction with supervision or mentorship received *was* associated with greater job satisfaction.^38,46^ Higher autonomy was beneficial,^26^ being associated with lower burnout and higher job satisfaction.^34,40,46^ Similarly, adequate resourcing was key; dissatisfaction with work-related resources, including remuneration, predicted higher burnout and depression levels, and lower job satisfaction.^26,34,37,38,47^

Most research examining organisational factors focused on workload. Large workloads with limited time frames were stressful,^26,34,38^ prompting burnout, lower job satisfaction, and higher depression scores.^27,30,34^ Conversely, satisfaction with leadership positively correlated with job satisfaction.^27,37,38,46^ Programme change could produce mixed effects, threatening supervisors’ mental health, but also offering vitality if change was embraced as an opportunity and challenge.^34,49^

Regarding the regulatory, business and payment environment, GP supervisors reported that being appreciated, acknowledged, and respected by one’s colleagues and organisation supported job satisfaction.^26,27^ Likewise, supervisors who favourably rated their hospital’s credentialling policies reported higher job satisfaction.^37^

At a sociocultural level, several studies examined the effects of the COVID-19 pandemic on supervisor wellbeing. Three-quarters (*n* = 233) of supervisors in one study felt that the pandemic had increased their work-related stress.^32^ Key wellbeing threats introduced by the COVID-19 pandemic included contracting and transmitting the virus, protective equipment shortages, reduced collegial engagement, and administrative overload.^25,29,32^ However, supervisors also noted positive changes, including increased time with loved ones and engaging in self-care activities, and greater solidarity among GPs.^29,32^

### Why supervisor wellbeing is important

Supervisors’ wellbeing was linked to two outcomes. The first was retention; supervisors reported that burnout and low wellbeing prompted decisions to quit their jobs.^26,35^ Similarly, low job satisfaction predicted supervisors’ intent to quit.^37^ Second, supervisors’ emotional wellbeing positively correlated with their self-reported teaching and research productivity and engagement, plus their self-reported clinical productivity.^25^ Indeed, in one study supervisors’ job satisfaction accounted for one-quarter of the variance in resident satisfaction with teaching quality.^46^

### How to support supervisors’ wellbeing

Recommendations to improve supervisor wellbeing targeted individual, organisational, and cultural factors. In some studies interventions were evaluated. Suggestions for individual interventions included mentoring (to bolster role sustainability),^35,39^ and offering job orientations to minimise new supervisors feeling overwhelmed.^39^ In one pilot programme, faculty met for eight consecutive weeks to share and reflect on their personal origin stories.^43^ Although participants’ burnout levels did not significantly change, participants viewed the programme positively, experiencing affirmation, validation, and enhanced long-term peer connectedness. Another study noted that less than one-fifth of supervisors reported accessing wellbeing support services, despite most being aware of their existence.^29^

Two studies evaluated organisational and cultural interventions. One organisation instituted an anonymous, repeated survey for supervisors to provide feedback on the workplace.^40^ After 12 months, supervisors reported a significant increase in their sense of workload control, while burnout and job-related stress levels showed medium, although non-significant, declines. Supervisors also identified minimal, non-significant benefits for job satisfaction and values alignment with leadership.^40^ Another institution embarked on a major cultural change initiative, including using a visual symbol and incorporating rituals to enhance the sense of identity within a residency programme. However, this change largely focused on trainees, leaving supervisors with less understanding of the changes.^33^

## Discussion

### Summary

This review consolidates the published literature regarding the wellbeing of family medicine and general practice supervisors, identifying 26 unique samples across 27 studies. No articles focused on conceptualisations of wellbeing. There was also limited consideration of the impact of supervisors’ wellbeing on their roles, or of interventions to support this group. Although risk and protective factors were explored more extensively, this largely concerned individual rather than organisational or systemic factors.

### Strengths and limitations

Several characteristics of the literature limited the present review’s strength. First, most studies were conducted in the USA, potentially limiting the applicability of findings to other countries with different training models. Future research in other settings would inform the transferability of the findings. Second, research is predominantly cross-sectional, impeding understanding of directionality in observed relationships. Future longitudinal studies will help address this. Third, although external factors (particularly sociocultural and regulatory factors) are likely important, there has been little consideration of these.^59–65^ Finally, much of the reviewed literature focused on burnout and poor wellbeing rather than ‘positive’ wellbeing (for example, thriving, flourishing). A better understanding of positive wellbeing has implications for future educational outcomes and workforce sustainability.

Further factors regarding the methodology of this review should be considered when viewing the above findings. First, the review was confined to GP supervisors, so applicability to other specialty supervisors remains unclear. Future literature reviews examining other specialty supervisors can address this gap. Additionally, date restrictions were not imposed on the literature, because this is the first review of its kind. More than one-third (N^studies^ = 10, 37%) of included studies were published over a decade ago limiting the relevance of these findings. Since one-third of included articles were published within the past 3 years, this area of research is attracting increasing attention. Only two studies evaluating interventions were identified, which impeded our ability to comment on strategies to support supervisors’ wellbeing. Finally, given the heterogeneity in the assessment of wellbeing, this limited the review’s ability to robustly synthesise findings. Greater conceptual clarity will help address this in future reviews.

### Comparison with existing literature

Regarding wellbeing conceptualisations, mapping the review’s findings against a model of GP trainee wellbeing highlights that key aspects of wellbeing have been overlooked (for example, relationships, personal–professional interactions).^55^ The heterogenous and piecemeal assessment of wellbeing observed in this review emphasises calls for strengthening theoretical understandings to enable more consistent and sophisticated research.^66–68^

The review highlighted that the risk and protective factors for GP supervisor wellbeing mirror those for GP trainees. As with GP trainees, sociodemographic factors produced largely mixed effects.^69–71^ Despite fewer external factors being explored, these findings were more consistent. Again, as with trainees,^72–74^ this review indicated that autonomy, collegial relationships, and resource availability are key determinants of supervisors’ wellbeing. Similarly, there was considerable overlap in the factors supporting job satisfaction for GP supervisors and GPs more broadly.^75,76^

To date, little attention has been paid to the potential consequences of supervisor wellbeing. The review’s findings on this point were therefore limited, but indicate that poor GP supervisor wellbeing is associated with increased supervisor turnover and reduced educational quality. This underscores the importance of supporting supervisor wellbeing.^1,2,77,78^

### Implications for research and practice

The primary purpose of this review was to determine knowledge gaps and inform research priorities within this area, especially regarding intervention development and evaluation. The risk and protective factors identified in the review suggest individual targets of building psychological flexibility and skills in establishing boundaries between work and personal time. The review also highlights that organisational interventions should build supervisors’ role autonomy and peer interaction.^13,74^ The greater consistency in the effects observed for external — rather than internal — risk and protective factors in this review is important. It reiterates that, although we need to continue supporting supervisor wellbeing at an individual level, emphasis must be placed on organisational and cultural interventions.^57,79^ These will have the added benefit of simultaneously supporting postgraduate medical trainees’ wellbeing. The larger body of literature on GP trainee interventions could guide intervention development for GP supervisors.^80^

In conclusion, this review consolidates the literature focused on GP supervisor wellbeing. Research examining risk and protective factors has largely focused on individual — particularly personal — factors, while organisational and cultural factors remain future research priorities. Little research has investigated interventions to enhance GP supervisor wellbeing. This review is timely and highlights research to support GP supervisors’ wellbeing into the future, which will ultimately enhance wellbeing among the medical profession.

## Data

The data extraction template is provided in Appendix 2. Extracted data from individual studies is not provided, since copyrighted articles are included in the data files.
